# Branch facial nerve trauma after superficial temporal artery biopsy: a case report

**DOI:** 10.1186/1752-1947-5-34

**Published:** 2011-01-26

**Authors:** Richard A Rison

**Affiliations:** 1 University of Southern California, Keck School of Medicine, Los Angeles County Medical Center, Los Angeles, California USA; Presbyterian Intercommunity Hospital, 12401 Washington Blvd., Whittier, California 90602, USA

## Abstract

**Introduction:**

Giant cell arteritis is an emergency requiring prompt diagnosis and treatment. Superficial temporal artery biopsy is the gold diagnostic standard. Complications are few and infrequent; however, facial nerve injury has been reported, leaving an untoward cosmetic outcome. This case report is to the best of our knowledge only the fourth one presented in the available literature so far regarding facial nerve injury from superficial temporal artery biopsy.

**Case presentation:**

A 73-year-old Caucasian woman presented for neurological evaluation regarding eyebrow and facial asymmetry after a superficial temporal artery biopsy for presumptive giant cell arteritis-induced cephalalgia.

**Conclusion:**

Damage to branches of the facial nerve may occur after superficial temporal artery biopsy, resulting in eyebrow droop. Although an uncommon and sparsely reported complication, all clinicians of various specialties involved in the care of these patients should be aware of this given the gravity of giant cell arteritis and the widespread use of temporal artery biopsy.

## Introduction

Giant cell arteritis (GCA) is a neurologic emergency requiring prompt diagnosis and treatment. For years, the gold standard diagnostic test has been superficial temporal artery biopsy (STAB). This procedure is generally well tolerated with infrequent complications. Facial nerve injury is a known but uncommon complication, with few reported cases.

## Case presentation

A 73-year-old Caucasian woman with a one-year history of headaches and left temporal tenderness developed an elevated erythrocyte sedimentation rate. She was started on prednisone and a few days later underwent a left-sided STAB as an outpatient. In the recovery room, she noted difficulty closing her left eye secondary to weakness. She also noted numbness along the left portion of her face. There were no complaints of blurry or double vision, difficulty swallowing or slurred speech. She did not complain of any weakness in her arms or legs. She did not have vertigo, tinnitus, diminished hearing or lapses of consciousness. She was told at the time that she may have had an adverse effect from the local anesthetic and was discharged home with eye drops.

The patient's medical history was remarkable for hypertension, hypercholesterolemia, a past transient ischemic attack, fibromyalgia and gout. She was taking levothyroxine, simvastatin, duloxetine, lisinopril, hydrochlorothiazide, clopidogrel and prednisone.

The patient presented for neurologic evaluation six weeks after the STAB. General physical examination revealed a blood pressure in her left arm (sitting position) of 110/70 mm Hg with a regular pulse. Her height was 5'3", and she weighed 152 lb. There were no carotid bruits or cardiac murmurs, and her lungs were clear to auscultation. She had a well-healed scar over the region where the left STAB had been done. Cranial nerve examination was remarkable for significant limitation of left eye closure (her eyelid could not adequately cover her sclera beneath her pupil). There was also diminished upward furrowing of her left brow with minimal mild and slow weakness seen in the left lower portion of her face, producing mild asymmetry. The right facial muscles were all completely intact. No facial synkinesis was witnessed. Facial sensation was intact in a bilateral V1 through V3 distribution to light touch and temperature. Her pupils were round, equal and symmetric to light. Visual fields were intact, and disc margins were sharp on funduscopic examination. All ocular movements were intact with conjugate gaze and without nystagmus. All muscles of mastication were intact. She had no hearing deficits, and visual inspection of the bilateral internal auditory canals did not reveal any hemorrhages, erythema, vesicles or exudates. Her palate elevated midline, and her gag reflex was intact. Her bilateral sternocleidomastoid and trapezius muscles all displayed intact strength, and her tongue protruded midline without any fasciculations or atrophy. The remainder of the neurologic examination revealed no focal motor, sensory, coordination, gait or reflex deficits. Her speech and mental status were both quite intact.

Investigations were significant for a magnetic resonance imaging study with and without contrast that revealed cerebral ischemic gliosis compatible with the patient's age without acute intracranial pathology. There were no abnormalities noted along the course of either cranial seventh nerve. Her left STAB incision did not show evidence of thrombus, inflammation or giant cells and hence was without evidence of temporal arteritis.

## Discussion

GCA is a medical emergency characterized by systemic inflammation and critical ischemia with early neuro-ophthalmic complications. It is the most common vasculitis seen in Western countries involving large- and medium-sized arteries with a predilection toward the cranial arterial vasculature. There is a female preponderance with increasing frequency as one ages. Permanent visual loss can occur in up to 20% of patients and is the best known and most feared complication. STAB remains the gold standard for diagnosis [1].

STAB is a widely performed procedure with relative safety. It is usually performed under local anesthesia in an office or same-day surgical setting [2]. The incidence of complications after STAB is quite low, with the majority of cases being temporary and minor [3]. Complications include incorrect or inadequate tissue sampling, bleeding, hematoma formation if the arterial ligature slips [4], scarring, infection, wound dehiscence, and rarely cerebral ischemia after the biopsy when the temporal artery provides essential collateral circulation in the case of severe ipsilateral carotid disease [3,5]. The latter complication of cerebral ischemia can be clinically distinguished from branch facial nerve trauma by observation of disproportionally increased facial motor involvement expected in upper motor neuron lesions (e.g., lower facial weakness).

The surgical technique and anatomy of STAB has been well described in the literature [6,7], and an additional complication may be eyebrow droop from damage to branches of the facial nerve if the incision is taken too close and parallel to the eyebrow [4]. Although this may be mentioned in standard STAB consent forms, there have only been three prior published reports of facial nerve injury after STAB, the last one of which was almost 10 years ago (Table 1). Slavin [8] and Bhatti and Taher [9] have published cases of eyebrow droop after STAB. Bhatti and Goldstein [3] reported on a 75-year-old woman with presumed GCA who developed frontalis muscle paralysis after the biopsy. Inadvertent direct injury to the branch facial nerve may occur because the surgical incision may be in a "danger zone", an anatomic area of potential injury in which the branches of the superficial temporal artery course within the superficial temporal fascia close to the temporal branches of the facial nerve that run beneath within a loose alveolar layer [3]. While in this region, the surgeon must take particular care not to dissect below the superficial temporal fascia (as may happen in difficult cases) using only gentle blunt maneuvers to separate the subdermal fatty layer and lose fascial attachments to isolate the superficial temporal artery [3]. The paucity of reported adverse outcomes, however, attests to the overall general safety of the standard and commonly used STAB sites. Even with anatomic variations, STAB still remains a quite safe procedure.

**Table 1:** Previously reported cases of branch facial nerve injury after superficial temporal artery biopsy

Slavin (1986) [8]	A 55-year-old woman with eyebrow droop after a superficial temporal artery biopsy
Bhatti and Taher (2000) [9]	A 63-year-old woman with partial facial paralysis after temporal artery biopsy

Bhatti and Goldstein (2001) [3]	A 75-year-old woman with facial nerve injury after superficial temporal artery biopsy

The mechanism of injury in the presented patient may have been a local branch facial nerve neuropraxia. Other possibilities include local hematoma formation (perhaps precipitated by clopidogrel, which was not held preoperatively). Vasculitis as a cause was thought to be less likely given the negative findings on the biopsy specimen. Fortunately, at one year later, the patient had complete resolution of her signs and symptoms without any adverse cosmetic outcome (making nerve section an unlikely mechanistic cause). She had no evidence of any permanent peripheral facial nerve damage.

Given the wide variety of specialties that perform STAB (dermatologists, ophthalmologists, general surgeons, vascular surgeons and plastic surgeons) along with the family practitioners, internists, rheumatologists and neurologists who follow these patients, it behooves all clinicians to be aware of this uncommon outcome, especially because it seems that complications are underreported in the literature. Having in mind these potential severe complications, STAB should only be performed by experienced hands.

## Conclusion

GCA and STAB are frequently multidisciplinary entities. Damage to branches of the facial nerve may occur after STAB. Although an uncommon and sparsely reported complication, all clinicians of various specialties involved in the care of these patients should be aware of this condition given the gravity of GCA and the widespread use of STAB.

## Abbreviations

GCA: giant cell arteritis; STAB: superficial temporal artery biopsy.

## Competing interests

The author declares that they have no competing interests.

## Consent

Written informed consent was obtained from the patient for publication of this case report. A copy of the written consent is available for review by the Editor-in-Chief of this journal.

## Authors' contributions

RAR performed the history and physical (including neurologic) examination and wrote the entire manuscript.
